# Erratum: Virus-like particles are efficient tools for boosting mRNA-induced antibodies

**DOI:** 10.3389/fimmu.2023.1183189

**Published:** 2023-03-21

**Authors:** Frontiers Production Office

**Affiliations:** Frontiers Media SA, Lausanne, Switzerland

**Keywords:** SARS-CoV-2, vaccine, virus-like particles, mRNA, COVID-19

An omission to the funding section of the original article was made in error. The following sentence has been added: “Open access funding was provided by the University Of Bern”.

The original version of this article has been updated.

